# Linc01748 regulates the prognosis and related mechanisms of gastric cancer by targeting miR-130a-5p

**DOI:** 10.1186/s41065-026-00634-5

**Published:** 2026-01-24

**Authors:** Hui Li, Zhiling Zhong, Yuhan Li, Meini Cen

**Affiliations:** 1 Medical School, Zhengzhou Sias University, Zhengzhou, 451100 China; 2 Gastroenterology, The Second Hospital of Dalian Medical University, Dalian, 116000 China; 3 College of Clinical and Basic Medicine, Institute of Basic Medicine, Shandong First Medical University & Shandong Academy of Medical Sciences, Jinan, 250117 China; 4 Guangxi Key Laboratory for Preclinical and Translational Research on Bone and Joint Degenerative Diseases, No. 18, Zhongshan 2nd Road, Youjiang District, Baise, 533000 China; 5 Pediatric Intensive Care Unit, The Affiliated Hospital of Youjiang Medical University for Nationalities, No. 18, Zhongshan 2nd Road, Youjiang District, Baise, 533000 China

**Keywords:** Gastric cancer, Prognosis, Linc01748, MiR-130a-5p, POU2F1

## Abstract

**Objective:**

The purpose of this study is to investigate the expression of linc01748 in gastric cancer (GC) tissues and cell lines, and to reveal the influence and potential mechanism of linc01748 on the biological behavior of GC cells.

**Methods:**

Linc01748 levels in GC were predicted through bioinformatics software. The proliferation, migration and invasion abilities of GC cells were respectively estimated by cell function tests. The RT-qPCR experiment was implemented to measure the levels of key proteins in epithelial-mesenchymal transition (EMT). The miRNAs regulated downstream of linc01748 were preliminarily screened through literature review and bioinformatics prediction. The relationship of miR-130a-5p to linc01748 was validated by dual-luciferase assay. The effect of the linc01748/miR-130a-5p axis on the expression of EMT-related genes was detected via in vitro cell experiments. MiR-130a-5p target genes were predicted using bioinformatics databases and verified by luciferase reporter gene experiments.

**Results:**

Linc01748 expression in GC tissues and cell lines is significantly upregulated and is associated with prognosis. Linc01748 promotes the proliferation, migration and invasion of GC and regulates the expression of key proteins in EMT. miR-130a-5p is lowly expressed in GC and can mediate the cancer-promoting effect of linc01748. POU2F1 is the target gene of the linc01748/miR-130a-5p axis, and its expression is negatively correlated with miR-130a-5p.

**Conclusion:**

Linc01748 is highly expressed in GC tissues and cell lines and is closely related to the prognosis. The linc01748/miR-130a-5p axis regulates POU2F1 expression, thereby facilitating the progression of GC and EMT.

## Introduction

Globally, the second leading cause of death due to cancer is gastric cancer (GC) [1]. On the basis of data from WHO, the number of new cases and deaths in 2020 from GC globally was 1.09 million and 769,000 respectively, accounting for 7.7% of all deaths related to malignant tumors [2]. Although targeted therapy has improved the treatment outcomes of most cancer patients, the heterogeneity of the GC microenvironment remains a major obstacle to treatment [3]. GC in early-stage often has no obvious signs or clinical manifestations. When patients experience upper abdominal discomfort, nausea, vomiting and other symptoms, it often indicates that the disease is in the advanced stage [4]. Generally, early-stage GC is mostly well-differentiated or moderately differentiated adenocarcinoma, while middle and advanced-stage GC is mostly poorly differentiated or undifferentiated adenocarcinoma [5]. Most people are confirmed at the middle or advanced stage, losing the best treatment chance. This leads to an unsatisfactory prognosis and 5-year survival rate for GC treatment [6]. Therefore, strengthening the research on the etiology and pathogenesis of GC is of extremely significant importance for early detection, diagnosis and treatment of GC in the future.

The prognosis of GC is influenced by the interaction of multiple factors. A multi-database study identified FAT4 mutation as an independent favorable prognostic factor, linked to higher tumor mutation burden and a pro-inflammatory tumor immune landscape [7]. This finding suggests that there may be other mechanisms that have not yet been discovered, especially those involving non-coding RNA, which can influence the prognosis of patients by reshaping the tumor microenvironment. Non-coding RNA is a kind of RNA that does not have the function of coding proteins, including lncRNA, miRNA, circRNA [8]. It is a hot topic in the epigenetic research of GC. LncRNA is classified as an RNA molecule with more than 200 nucleotides and no protein-coding ability, mostly transcribed from different regions of the genome by RNA polymerase II [9, 10]. Although LncRNA does not have the ability to encode proteins, research has shown that lncRNA regulates genes at the transcriptional level, post-transcriptional level, translation level and other levels [11]. They can act as bait, activator, guide or scaffold between interacting proteins [12]. The carcinogenic mechanism of GC is a complex process, during which abnormal regulation of lncRNA is widespread. For instance, H19, located on chromosome 11p15.5, has been reported to have increased levels in GC tissues and AGS cells [13]. The highly expressed HOTAIR in GC is significantly connected with tumor stage, lymphatic metastasis and poor survival [14]. Linc01748 is a newly discovered gene related to human tumors [15–17]. In the GEO database, linc01748 was enhanced in GC tissues, and it was also found to be increased in GC in the LnCAR database. However, the mechanism of this gene in GC remains unclear at present.

Competitive endogenous RNA (ceRNA) is usually involved in the regulatory mechanism of cancer gene expression [18]. The competitive binding of lncRNA as ceRNA to miRNA to construct specific lncRNA-miRNA-mRNA networks are the research warm spots in the field of oncology [19]. For instance, LINC00355 can promote the growth of GC cells via adjusting the miR-15a-5p/PHF19 axis as a ceRNA [20]. Therefore, in this study, we aimed to investigate the role and mechanism of linc01748 in GC pathogenesis. Our central hypothesis is that linc01748 promotes GC malignancy by antagonizing the tumor-suppressive function of miR-130a-5p. To test this hypothesis, we designed experiments to confirm the oncogenic functions of linc01748 in vitro, to verify the direct interaction between linc01748 and miR-130a-5p, and to elucidate the functional consequences of this interaction on GC cell phenotypes.

## Materials and methods

### Research subjects and groups

The GC tissue samples were taken from the specimen bank of patients who underwent surgical treatment in The Affiliated Hospital of Youjiang Medical University for Nationalities. Tissues that did not receive radiotherapy and chemotherapy before the operation and non-cancerous tissues more than 5 cm away from the tumor tissue were collected, totaling 97 groups. Meanwhile, the clinicopathological information of these patients was extracted. All patients were diagnosed with gastric cancer through preoperative examinations such as B-ultrasound, biopsy and CT, and the postoperative pathology confirmed it. This study has been reviewed and authorized by the ethics committee, and all the subjects have given informed consent.

### Prognosis analysis

Patients joined a 5-year follow-up. The follow-up methods are mainly outpatient visits or telephone communication. The completion of the patient’s surgery was taken as the starting time of the follow-up, and 5 years after the operation was taken as the end time of the follow-up. The follow-up frequency was once every 3 months in the first 2 years, once every 6 months in the middle 2 years, and once a year in the last year. Given that there is no pre-defined clinical cut-off value for linc01748, the median provides an objective and data-based threshold. Therefore, patients were stratified into low linc01748 expression group (*n* = 48) and high linc01748 expression group (*n* = 49) based on the median expression value. In addition, to evaluate the robustness of the prognostic model, patients were divided into the high linc01748 expression group (*n* = 24) and the low linc01748 expression group (*n* = 24) using top-bottom quartiles. The survival conditions of each group were statistically analyzed, and the Kaplan-Meier survival curve was plotted.

### Experimental cells

Normal gastric cell line (GES-1), and human gastric adenocarcinoma cell lines (HGC-27, BGC-823, MKN-74 and AGS) were all acquired from the cell bank of the Chinese Academy of Sciences. GES-1 is an immortalized gastric mucosal cell line established by infecting 9-month fetal gastric epithelial cells with SV40 virus, serving as a non-tumorigenic control. Although immortalized, GSE-1 cells retain key characteristics of normal gastric epithelium and have been widely used as a normal control in GC research [21]. AGS used Ham’s F-12 medium. GSE-1, BGC-823 and MKN-74 were in RPMI 1640 medium. HGC-27 was in DMEM medium. 10% FBS and 1% penicillin/streptomycin need to be added to the culture medium of all cells. All the above-mentioned cells were grown in a cell culture incubator at 37℃ and 5%CO_2_. Conventional subculture was performed when the cell fusion reached 80%. AGS and MKN-74 cells in good growth condition were selected for transfection. Under the induction of the transfection reagent Lipofectamine 2000, si-linc01748 and miR-130a-5p mimic or inhibitor, as well as their respective negative controls, were introduced into the cells.

### CCK8 assay

After trypsinization, the harvested cells were resuspended and seeded into 96-well plates at a density of 2 × 10^3^ cells/well. To assess cell viability at various time points (0, 24, 48, and 72 h), 10µL of CCK8 solution was directly added to each well. The plates were subsequently incubated for 2 h at 37℃ in a humidified 5% CO₂ atmosphere. Finally, the OD value at 450 nm was determined through a microplate reader.

### Transwell assay

The transfected cells were digested with trypsin, resuspended and counted in serum-free medium. Inoculate 50µL of cells into the upper layer of Transwell chambers and place them at 37 °C for 0.5–2.5 h. Add 800µL of complete culture medium to the lower chamber of Transwell. After 12 h of culture, aspirate the old culture medium and rinse the chamber with PBS buffer. Be careful to avoid touching the lower surface of the chamber to prevent cell detachment. Add 0.2% ammonium oxalate crystal violet staining solution in the lower room and stain at 37 °C for 20 min. After staining, the chamber was washed again with PBS, dried, and then observed under a microscope. Five fields of view were randomly selected for photography and counted using image J.

### RT-qPCR

Total RNA was obtained from GC tissues, normal tissues and cells by the Trizol method. 500µL of TRIzol reagent was added to the sample and mixed well, left at room temperature for 10 min. Then add chloroform, shake vigorously for 15 s, and centrifuge at 12,000 rpm for 15 min at 4 °C. Transfer the upper aqueous phase to a new centrifuge tube, add an equal volume of isopropyl alcohol, gently mix well, and let it stand at room temperature for 10 min. After re-centrifugation, add 500µL of 75% ethanol to wash the RNA precipitate, and then add an appropriate amount of RNase-free water to dissolve the RNA. The concentration and purity of the isolated total RNA were quantified using a NanoDrop 2000 spectrophotometer (Thermo Fisher Scientific, USA). Only RNA samples with an A260/A280 ratio between 1.8 and 2.1 and an A260/A230 ratio greater than 2.0 were used for subsequent experiments. The RNA integrity was further verified by 1% agarose gel electrophoresis, displaying clear 28 S and 18 S ribosomal RNA bands. RNA was reverse transcribed into cDNA via the PrimeScript first-strand cDNA Synthesis Kit (Takara, Dalian, China). PCR reactions were carried out using the Bio-Rad real-time PCR system and the SYBR Green kit. The amplification parameters are as follows: the initial denaturation is sustained at 95℃ for 30 s, followed by 40 cycles of denaturation at 95℃ for 30 s, annealing at 60℃ for 30 s, and extension at 72℃ for 30s. Using GAPDH and U6 as internal controls, the relative levels of linc01748, miR-130a-5p, POU2F1, E-cadherin and N-cadherin were detected by the 2^−△△Ct^ method.

Linc01748: (forward) 5’-TGCTACATGGGTAAGGGAGGG-3’,

(reverse) 5’-TTCTGGTAGACGCACTCGTCCT-3’;

miR-130a-5p: (forward) 5’-CCAGGGCTTTTCAAAAATGA-3’,

(reverse) 5’-CCGATCCAATCTGTTCTGGT-3’;

GAPDH: (forward) 5’-GGGAGCCAAAAGGGTCAT-3’,

(reverse) 5’-GAGTCCTTCCACGATACCAA-3’;

U6: (forward) 5’-AGAGAAGATTAGCATGGCCCCTG-3’,

(reverse) 5’-ATCCAGTGCAGGGTCCGAGG-3’;

POU2F1: (forward) 5’-ACGCACCAGCATAGAGACCAACAT-3’,

(reverse) 5’-TCACAGCAGCACTGGTTAAAGGGA-3’;

E-cadherin: (forward) 5’-TCCTCGCCCTGCTGATTCTG-3’,

(reverse) 5’-CTGGTCTTCTTCTCCACCTCCTTC-3’;

N-cadherin: (forward) 5’-AGCTCCATTCCGACTTAGACA-3’,

(reverse) 5’-CAGCCTGAGCACGAAGAGTG-3’.

### Western blot

Total cellular proteins were extracted using RIPA lysis buffer. After protein quantification was completed by the BCA method, denaturation treatment was carried out, and then SDS-PAGE electrophoresis was used for separation. The separated proteins were transferred onto PVDF membranes and blocked with skimmed milk. Subsequently, the primary antibody was incubated overnight at 4 °C (including N-cadherin, E-cadherin, and GAPDH), and the secondary antibody was incubated at room temperature for 1 h the next day. Finally, ECL development was carried out, and the gray values of different bands were analyzed using Image J software.

### Luciferase assays

The WT or MUT linc01748 and POU2F1 sequences were cloned into the psi-CHECK2 reporter vector to construct the luciferase reporter gene vector, WT-linc01748 and MUT-linc01748, WT-POU2F1 and MUT-POU2F1. AGS and MKN-74 cells were seeded onto 96-well plates at a density of 1 × 10^4^ cells. When the cells reached 80% of the fusion degree, the miR-130a-5p mimics or inhibitors and their negative controls were co-transfected with the above-mentioned reporter gene vectors respectively. The luciferase activity was detected 48 h later.

### RNA pull-down assay

To verify the direct interaction between linc01748 and miR-130a-5p, we conducted an RNA Pull-down experiment, using biotin-labeled linc01748 wild-type (WT) probes, binding site mutated (MUT) probes, and non-specific Control (Control) probes. These probes were incubated with streptavidin magnetic beads for binding. Then, this bead-probe complex is co-incubated with cell lysates to allow the formation of RNA complexes between linc01748 and potential binding molecules such as miR-130a-5p in the cells. Next, magnetic separation and multiple stringent washes are performed to remove non-specifically bound components. Finally, RNA is eluted from the beads using TRIzol and extracted, and the level of miR-130a-5p is specifically detected by RT-qPCR.

### Bioinformatics analysis

1. NCBI: The differentially expressed genes in GC were screened from the dataset GSE224056 of the National Center of Biotechnology Information (NCBI) database. The criteria were: the absolute value of log2Fold Change ≥ 1, and the *p* value < 0.05.
2. LnCAR: The differential expression of LINC00543 in GC was analyzed online by lnCAR (https://lncar.renlab.org/).
3. Prediction of miRNAs interacting with linc01748 using LncBase and ENCORI databases.
4. Target genes of miR-130a-5p were hunted using the miRDB, ENCORI, miRWalk and Targetscan databases, and the prediction results were used to construct Venn diagram to take the overlapping target genes for subsequent studies.
5. Protein-protein network analysis was conducted on the target genes of overlapping miR-130a-5p predicted by the database using STRING.
6. GO and KEGG pathway enrichment analysis of overlapping target genes using online database (http://www.bioinformatics.com.cn/login/).

### Data analysis

Data was processed using SPSS 22.0 and GraphPad Prism 7.0 software. Measurement data were expressed as mean ± SD. Independent sample t-tests or paired t-tests were used for two-group comparisons, and one-way ANOVA was used for multi-group comparisons. Counting data was denoted as n, and the Chi-square test is used for comparison between the two groups. The analysis of influencing factors was conducted using the Cox regression analysis method. A *p* value < 0.05 is considered significant.

## Results

### The expression of linc01748 is upregulated in GC

**Fig. 1:**
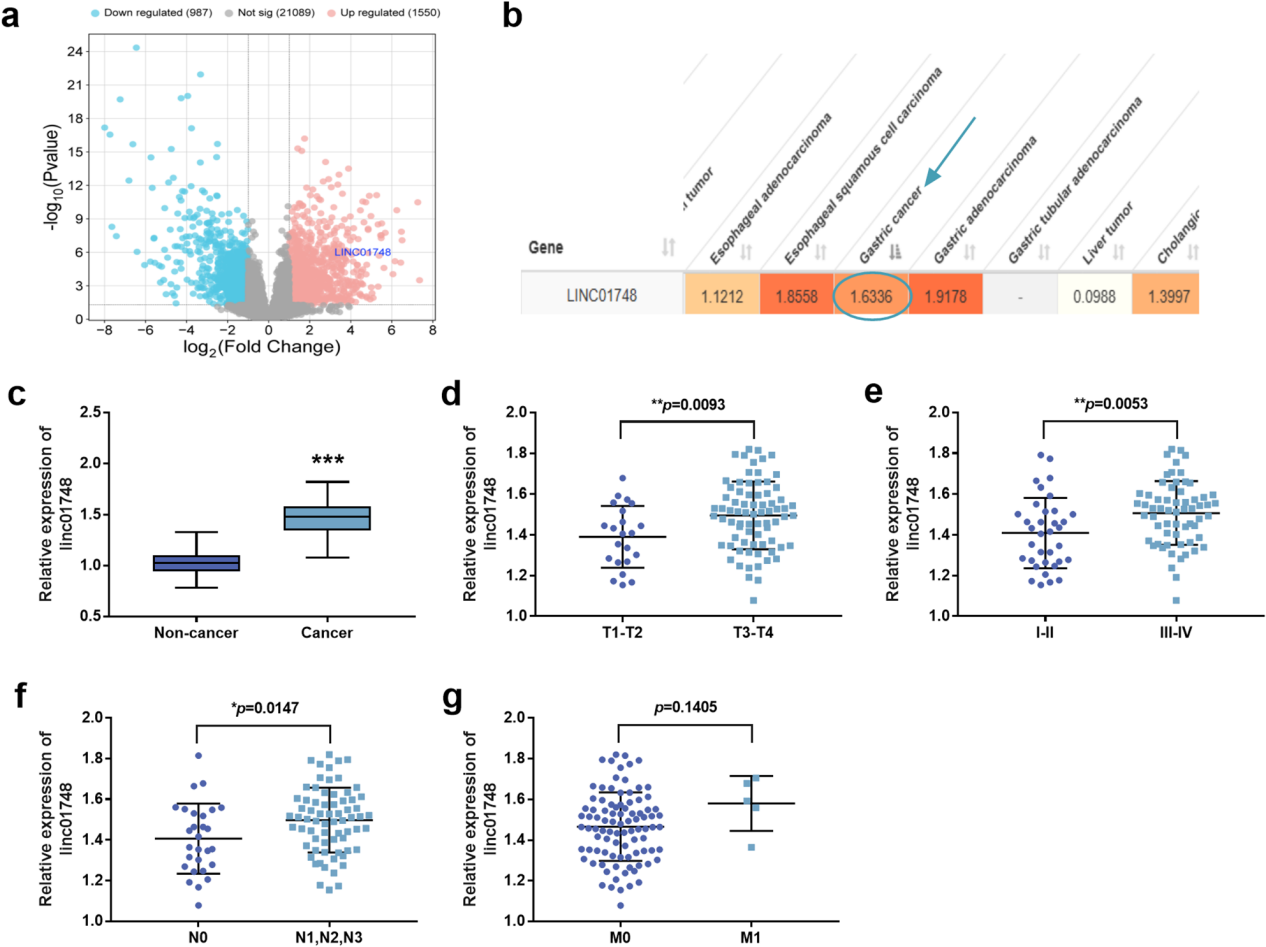
The expression level of linc01748 in gastric cancer. **a**. Linc01748 was upregulated in the tumor tissues of patients with gastric cancer in the dataset GSE224056. **b**. Linc01748 is upregulated in gastric cancer tissues in the LnCAR database. **c**. The expression of linc01748 in cancerous tissues increased in comparison to non-cancerous tissues. **d**. The linc01748 levels in patients with infiltration depth T3 and T4 were higher than those in T1 and T2. **e**. The linc01748 of patients with TNM III-IV is higher than that of patients with I-II. **f**. The linc01748 of patients with lymphatic metastasis N1, N2, and N3 was significantly higher than that of patients with N0. **g**. The expression of linc01748 in patients with distant metastasis at stage M1 showed no significant difference from that in patients at stage M0. ^***^p < 0.001, ^**^p < 0.01, ^*^p < 0.05

Analysis of the dataset GSE224056 demonstrated that linc01748 was upregulated in GC tissues compared with non-cancerous tissues adjacent to tumors (refer to Fig. 1a). linc01748 was screened out from the LnCAR database and it was found that its expression increased in GC tissues (refer to Fig. 1b). After analyzing 97 pairs of matched GC tissues and adjacent non-cancerous tissues in this study, it was indicated that the linc01748 level was significantly enhanced in GC tissues in comparison to non-cancerous tissues (Fig. 1c, *p* < 0.001). The expression of linc01748 in tissues with tumor invasion depths of T3/T4 is higher than that in T1/T2 (Fig. 1d, *p* < 0.01). Linc01748 expression in GC tissues at clinical stages III-IV is higher than that in I-II (Fig. 1e, *p* < 0.01). The expression level of linc01748 in GC tissues with lymphatic metastasis of N1, N2, and N3 is higher than that in N0 tissues (Fig. 1f, *p* < 0.05). No association was found between the difference in linc01748 levels and distant metastasis (Fig. 1g, *p* > 0.05).

### Relationship between the expression of linc01748 in GC tissues and clinicopathology

GC patients were divided into the high-expression group (*n* = 49) and the low-expression group (*n* = 48) based on the median expression level of linc01748. The Chi-square test was utilized to analyze the correlation between the level of linc01748 and the clinicopathological characteristics. As shown in Table 1, it was found that high expression of linc01748 was significantly correlated with tumor size, TNM stage, lymphatic metastasis and infiltration depth (*p* < 0.05). In addition, there were no statistically significant differences between the two groups in terms of gender, age, distant metastasis, and previous treatment history (*p* > 0.05).

**Table 1:** Relationship between linc01748 expression with clinicopathological variables

Variables	Patients (*n* = 97)	Linc01748		*p* value
		Low (*n* = 48)	High (*n* = 49)	
Gender				0.525
Female	34	15	19	
Male	63	33	30	
Age (years)				0.541
< 60	42	19	23	
≥ 60	55	29	26	
Size (cm)				**0.038**
< 5	38	23	14	
≥ 5	59	25	35	
TNM				**0.006**
I-II	35	24	11	
III-IV	62	24	38	
Lymphatic metastasis				**0.026**
N0	28	19	9	
N1, N2, N3	69	29	40	
Distant metastasis				0.362
M0	92	47	45	
M1	5	1	4	
Infiltration depth				**0.016**
T1, T2	22	16	6	
T3, T4	75	32	43	
Previous treatment history				0.312
Yes	46	20	26	
No	51	28	23	

A *p* value < 0.05 indicates a significant difference

*Abbreviations*: *TNM* tumor node metastasis

### Linc01748 and GC survival

**Fig. 2:**
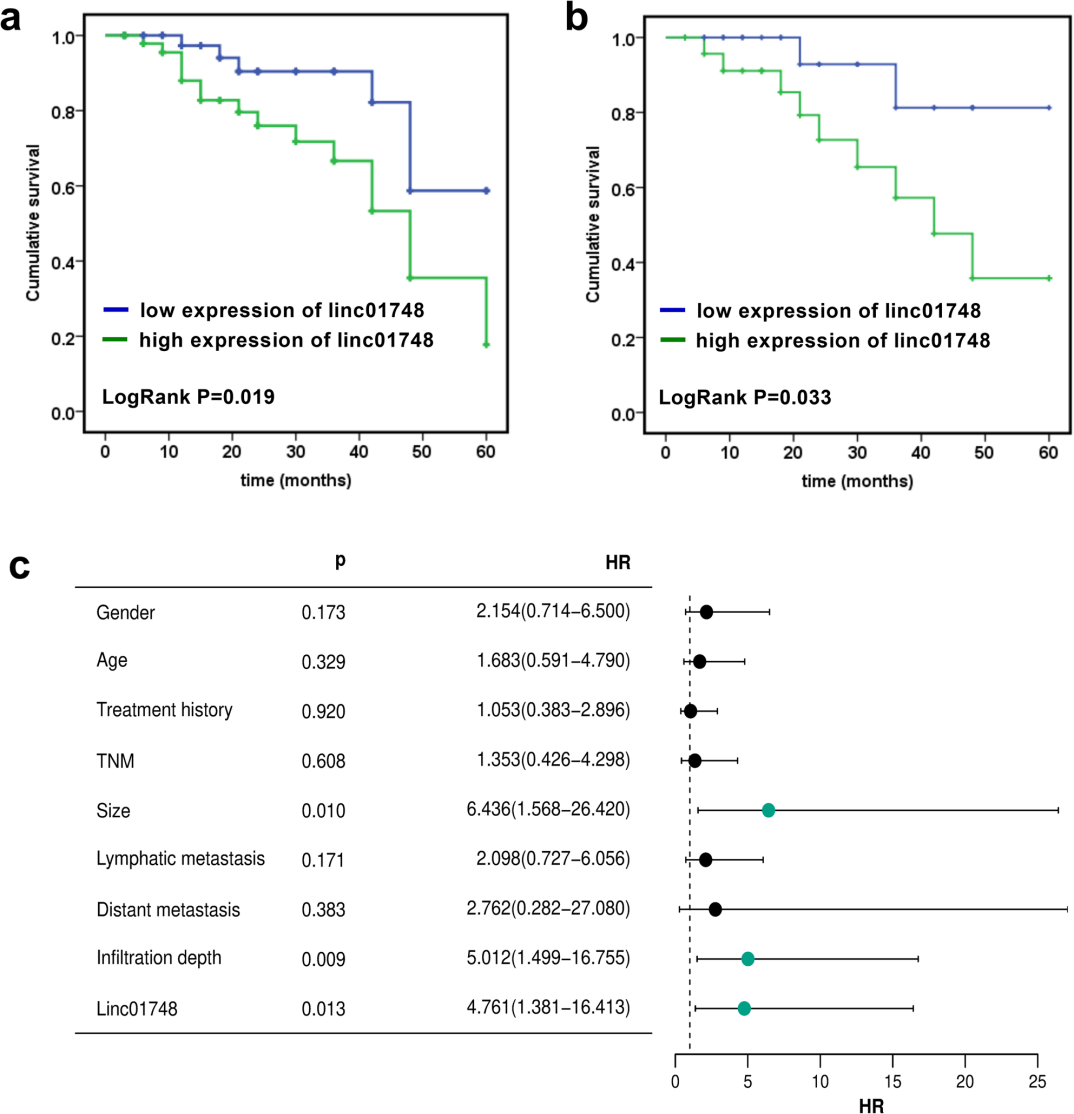
Survival analysis. **a**. Kaplan-Meier curve and log rank analysis (Group based on the linc01748 median value). **b**. Kaplan-Meier curve and log rank analysis (Group based on the top vs. bottom quartiles of linc01748 expression levels). **c**. Cox regression analysis and forest diagram

During the 5-year follow-up, 22 patients died. According to the median grouping, there were 16 cases in high-expression group and 6 in low-expression group. According to the top and bottom quartiles, there were 9 cases in the high-expression group and 2 cases in the low-expression group. The findings demonstrated that patients with high expression of linc01748 had a lower survival rate (Fig. 2a-b, *p* < 0.05). Multivariate Cox regression analysis exhibited that tumor size, infiltration depth, and the expression of linc01748 were significantly associated with the survival of GC patients (Fig. 2c, *p* < 0.05).

###  Linc01748 promotes the malignant phenotype of GC cells

**Fig. 3:**
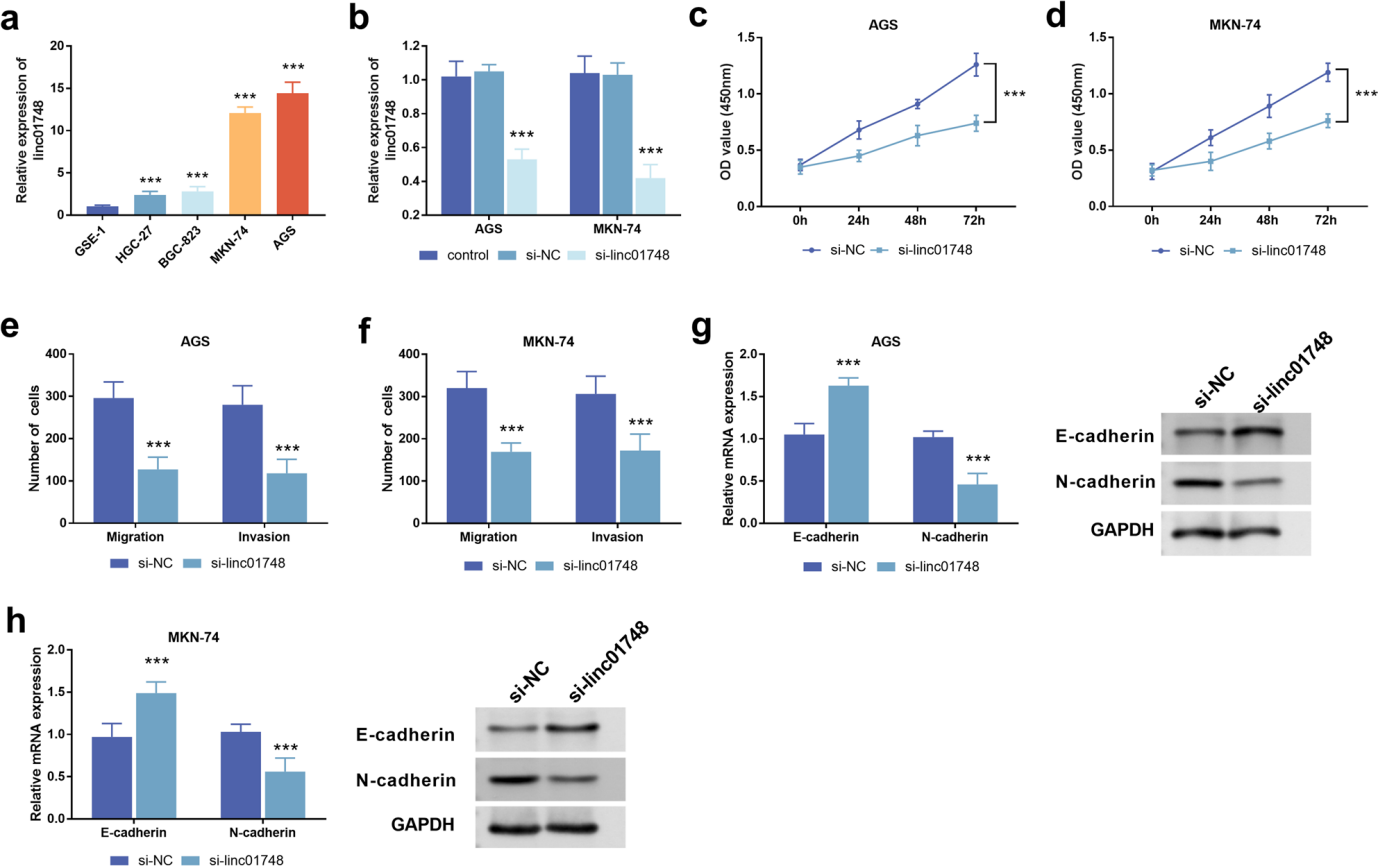
The effect of linc01748 on gastric cancer cell lines. **a**. The expression level of linc01748 in normal gastric cells and cancer cells. **b**. Transfection of si-linc01748 can down-regulate the expression of linc01748 in AGS and MKN-74 cells. **c**-**d**. Down-regulation of linc01748 can inhibit cell viability. **e**-**f**. Down-regulation of linc01748 inhibited cell migration and invasion. **g**-**h**. The RT-qPCR experiment and Western blot jointly demonstrated that inhibiting linc01748 can significantly suppress epithelial-mesenchymal transition. ^***^p < 0.001

Compared with GSE-1, linc01748 level was upregulated in HGC-27, BGC-823, MKN-74 and AGS, especially in MKN-74 and AGS (Fig. 3a, *p* < 0.001). Therefore, in subsequent experimental studies, AGS and MKN-74 were regarded as GC model cells. By transfecting si-linc01748 to knock down the expression of linc01748, we realized that linc01748 was markedly reduced in AGS and MKN-74 cells (Fig. 3b, *p* < 0.001). The results of the CCK8 experiment indicate that knocking down linc01748 is effective in inhibiting the proliferation ability of AGS and MKN-74 (Fig. 3c-d, *p* < 0.001). Transwell assays conveyed the message that the migration and invasion abilities of GC cell lines are weakened after silencing linc01748 (Fig. 3e-f, *p* < 0.001). The key proteins of epithelial-mesenchymal transition (EMT) were detected using RT-qPCR and western blot. The results displayed that in the two GC cell lines, after knocking down linc01748, E-cadherin increased, while N-cadherin decreased (Fig. 3g-h, *p* < 0.001).

### Linc01748 specifically binds to miR-130a-5p

**Fig. 4:**
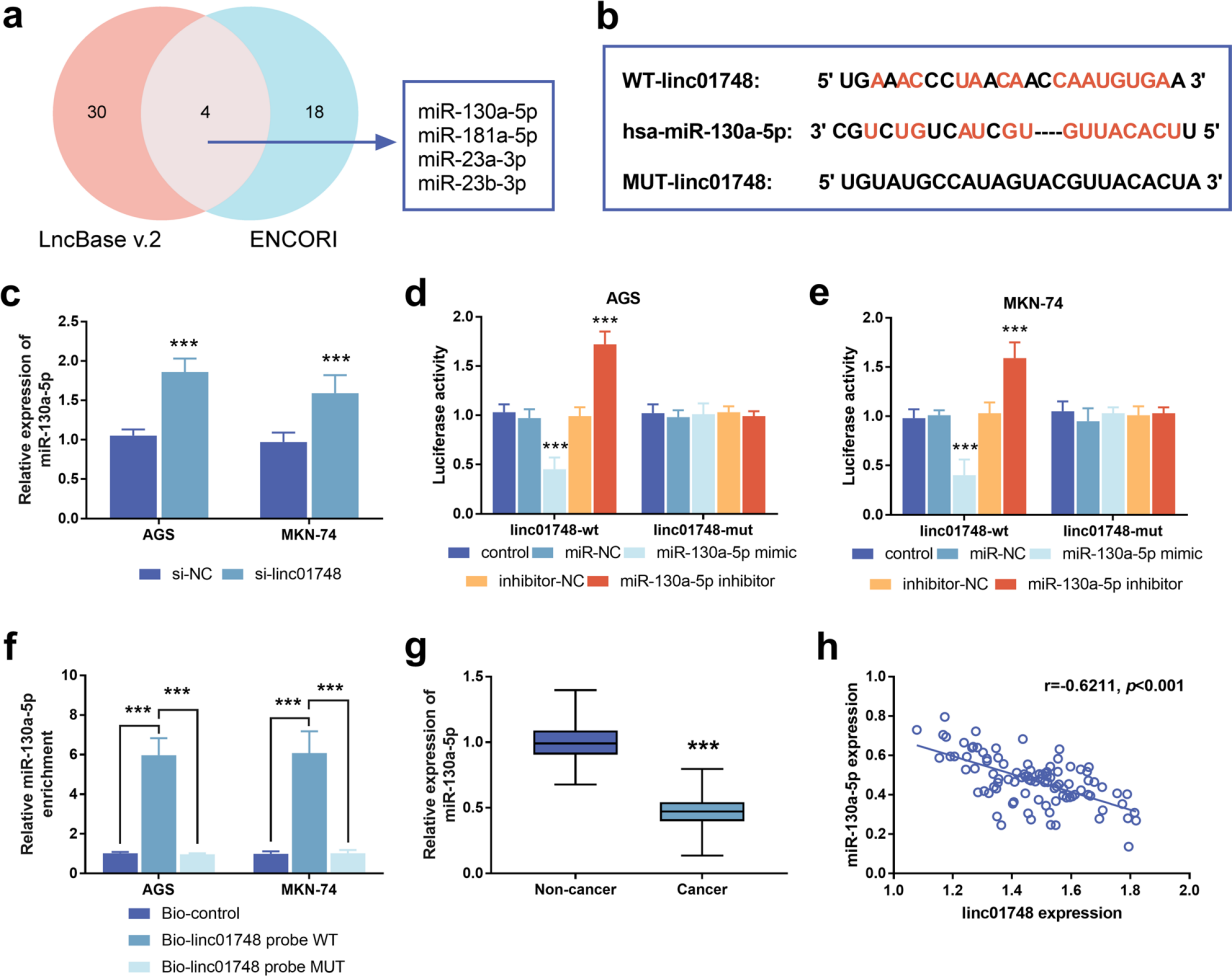
miR-130a-5p targets and binds to linc01748. **a**. The Venn diagram constructed using miRNAs that interact with linc01748 predicted by the LncBase v.2 database and the ENCORI database. **b**. The binding site of linc01748 and miR-130a-5p. **c**. Inhibition of linc01748 can increase the expression of miR-130a-5p in tumor cells. **d**-**e**. Dual-luciferase assay verified the interaction between linc01748 and miR-130a-5p. **f**. RNA pull-down experiment was used to verify the specific binding of linc01748 to miR-130a-5p. **g**. The expression miR-130a-5p is increased in tumor tissues. **h**. The expression of miR-130a-5p in the tumor tissue was negatively correlated with linc01748. ^***^p < 0.001

By taking the intersection of the prediction results of the LncBase v.2 database and the ENCORI database, four miRNAs related to linc01748 were obtained. According to the association with GC occurrence, miR-130a-5p was selected for study (refer to Fig. 4a). The potential binding sequence of miR-130a-5p and linc01748 is illustrated in Fig. 4b. The expression of miR-130a-5p was observed to increase after knocking down linc01748 in AGS and MKN-74 (Fig. 4c, *p* < 0.001). The luciferase reporter assay showed that the luciferase activity of the interaction between linc01748-wt and miR-130a-5p in the GC cell line was significantly decreased (Fig. 4d-e, *p* < 0.001). As shown in Fig. 4f, compared with the control probe, the linc01748 probe WT can specifically enrich miR-130a-5p (*p* < 0.001). In contrast, the enrichment ability of the MUT probe was significantly weakened, and its enrichment level was comparable to the background level of the control group. This result proves that linc01748 can directly bind to miR-130a-5p in a sequence-specific manner. The detection of 97 pairs of GC tissues and adjacent non-cancerous tissues revealed that miR-130a-5p was down-regulated in GC tissues **(**Fig. 4g, *p* < 0.001). Pearson test was conducted to analyze the correlation of linc01748 and miR-130a-5p in GC tissues, and it was found that the latter was negatively correlated with the former (Fig. 4h, *p* < 0.001).

### Linc01748/miR-130a-5p regulates the malignant phenotype of GC cells

**Fig. 5:**
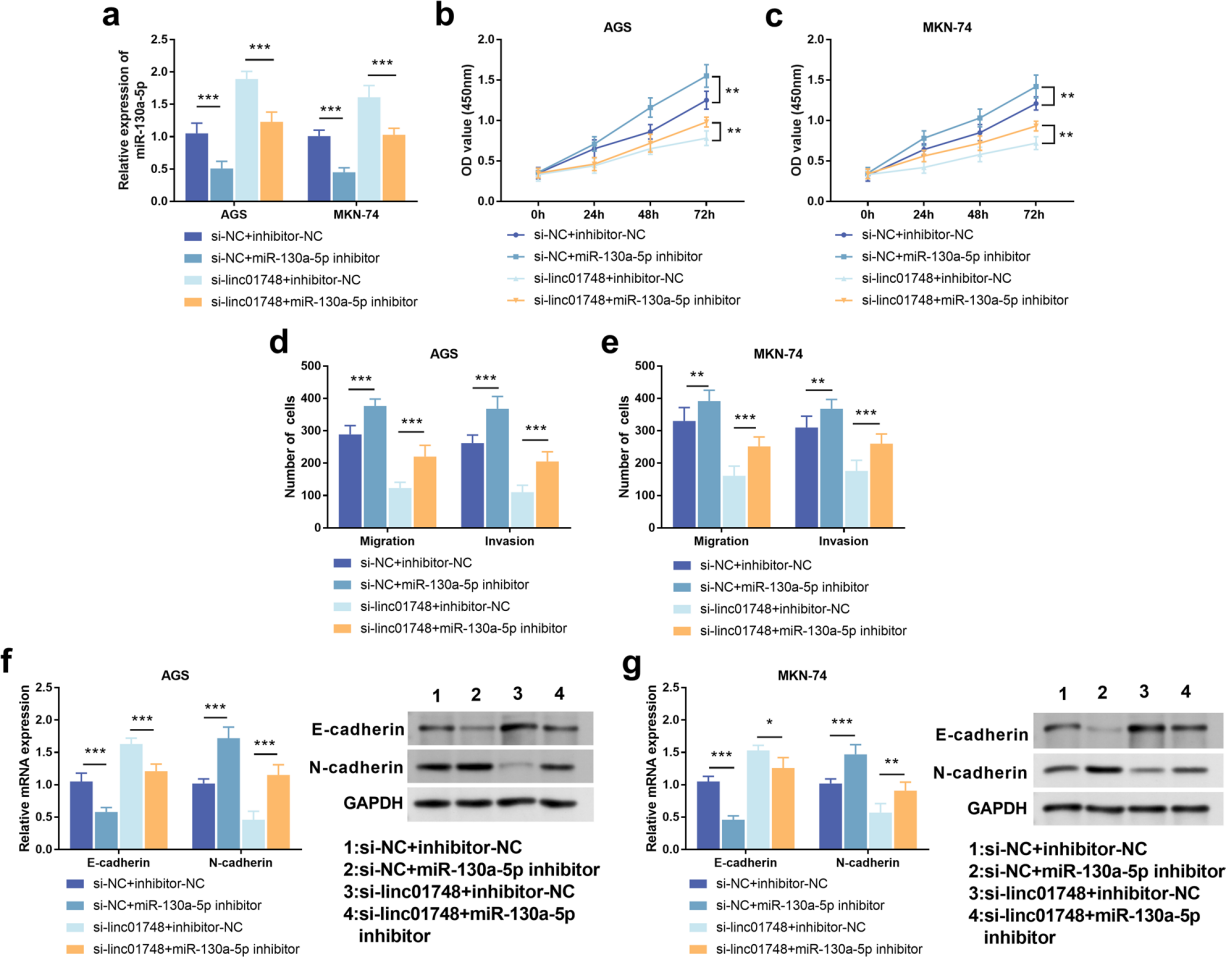
Linc01748/miR-130a-5p regulates the proliferation, migration, invasion and epithelial-mesenchymal transition of gastric cancer. **a**. The expression level of miR-130a-5p in AGS and MKN-74 cells after co-transfection. **b**-**c**. Co-regulate the effects of linc01748 and miR-130a-5p on the proliferation of AGS and MKN-74 cells. **d**-**e**. The Transwell assay verified the effect of co-transfection on migration and invasion. **f**-**g**. The effect of co-transfection on the expression of epithelial and mesenchymal markers was detected by RT-qPCR and Western blot assays. ^***^p < 0.001, ^**^p < 0.01, ^*^p < 0.05

After transfection of miR-130a-5p inhibitor in AGS and MKN-74, it was found that miR-130a-5p in the si-NC group decreased, and the elevated miR-130a-5p after knockdown of linc01748 was also downregulated with the addition of miR-130a-5p inhibitor (Fig. 5a, *p* < 0.001). CCK8 experiment demonstrated that miR-130a-5p effectively inhibited the proliferation of GC cells, and after suppressing miR-130a-5p, the inhibitory action of linc01748 silencing on cell proliferation could be weakened (Fig. 5b-c, *p* < 0.01). The Transwell assay confirmed that knockdown of miR-130a-5p could effectively promote the invasion and migration abilities of gastric cancer cells. Similarly, knockdown of miR-130a-5p could also partially overturn the inhibitory effect on gastric cancer cells after knockdown of linc01748 (Fig. 5d-e, *p* < 0.01). Besides, after inhibiting miR-130a-5p alone, both RT-qPCR and Western blot experiments jointly showed a decrease in E-cadherin, while an increase in N-cadherin. However, after knocking down linc01748, inhibiting miR-130a-5p could reduce the inhibitory effect of knocking down linc01748 on EMT (Fig. 5f-g, *p* < 0.05).

### Bioinformatics analysis of miR-130a-5p

**Fig. 6:**
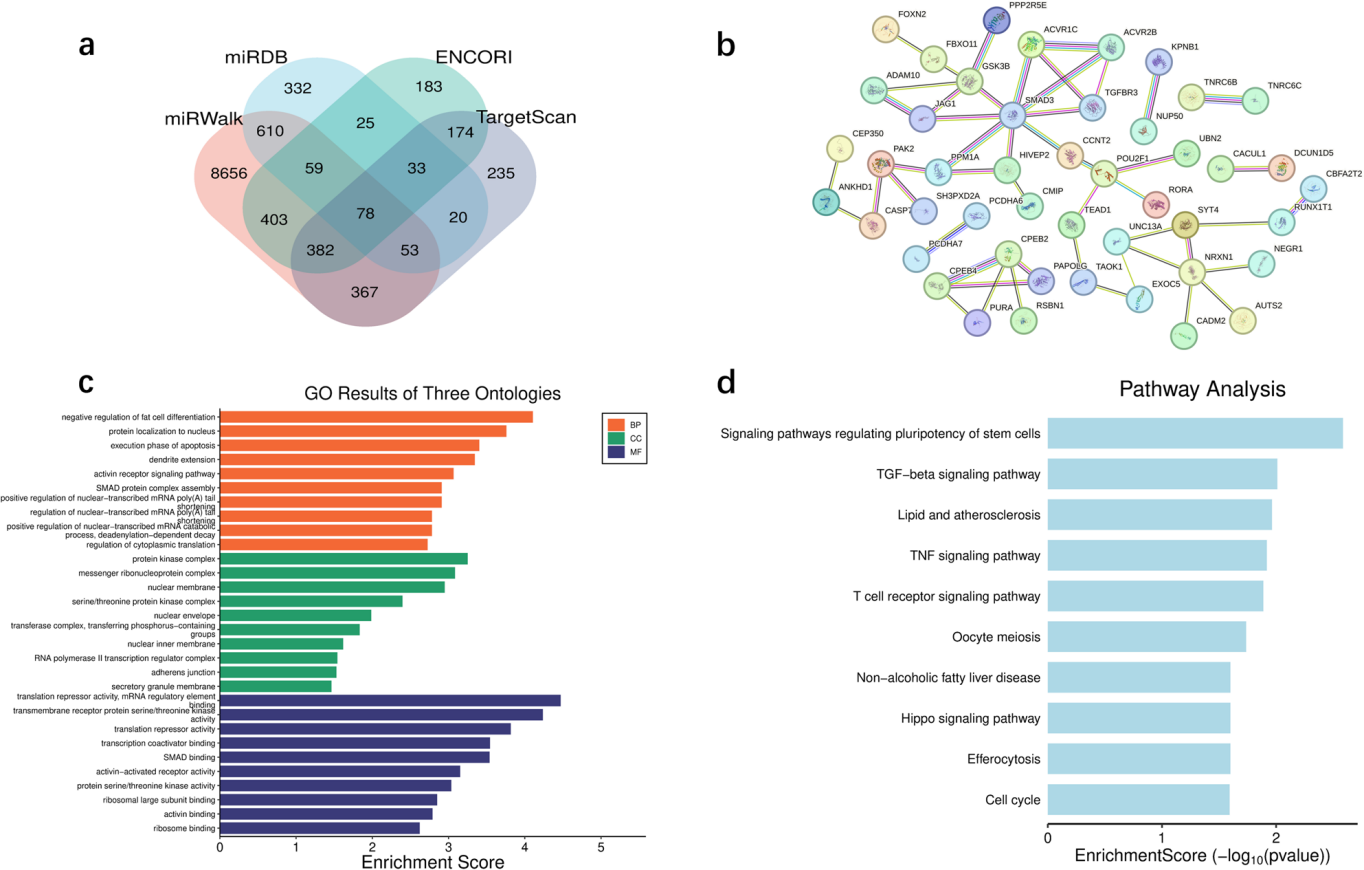
Bioinformatics analysis of downstream target genes of miR-130a-5p. **a**. Venn diagram of the downstream target genes of miR-130a-5p predicted by the database. **b**. The protein-protein interaction network of overlapping target genes. **c**. GO analysis of overlapping target genes. **d**. KEGG pathway enrichment analysis of overlapping target genes

The Venn diagram constructed from the prediction results of the four databases is exhibited in Fig. 6a, with 78 overlapping target genes. The PPI network diagrams of these target genes are shown in Fig. 6b. Table 2 exhibited the top 10 hub proteins related to miR-130a-5p. Figure 6c showed the GO analysis of overlapping target genes. Regarding biological processes, these genes are enriched in the stages of negative regulation of adipocyte differentiation and apoptosis execution. For cellular components, genes are gathered on protein kinase complexes and nuclear membranes. For molecular function, genes are enriched in the activity of translation suppressors. KEGG analysis illustrated that the signaling pathways enriched by these target genes were involved in the cell cycle, TNF/TGF-β/Hippo signaling pathways (refer to Fig. 6d).

**Table 2:** The top ten proteins in terms of the number of nodes in the PPI network

Protein	Full name	Node degree
SMAD3	Mothers against decapentaplegic homolog 3	8
GSK3B	Glycogen Synthase Kinase 3 Beta	5
NRXN1	Neurexin 1	5
CPEB2	Cytoplasmic polyadenylation element binding protein 2	4
POU2F1	POU class 2 homeobox 1	4
ACVR1C	Activin A receptor, type IC	3
ACVR2B	Activin A receptor, type IIB	3
CPEB4	Cytoplasmic polyadenylation element binding protein 4	3
HIVEP2	Human immunodeficiency virus type I enhancer binding protein 2	3
JAG1	Jagged1	3

### POU2F1 is a downstream target gene of miR-130a-5p

**Fig. 7:**
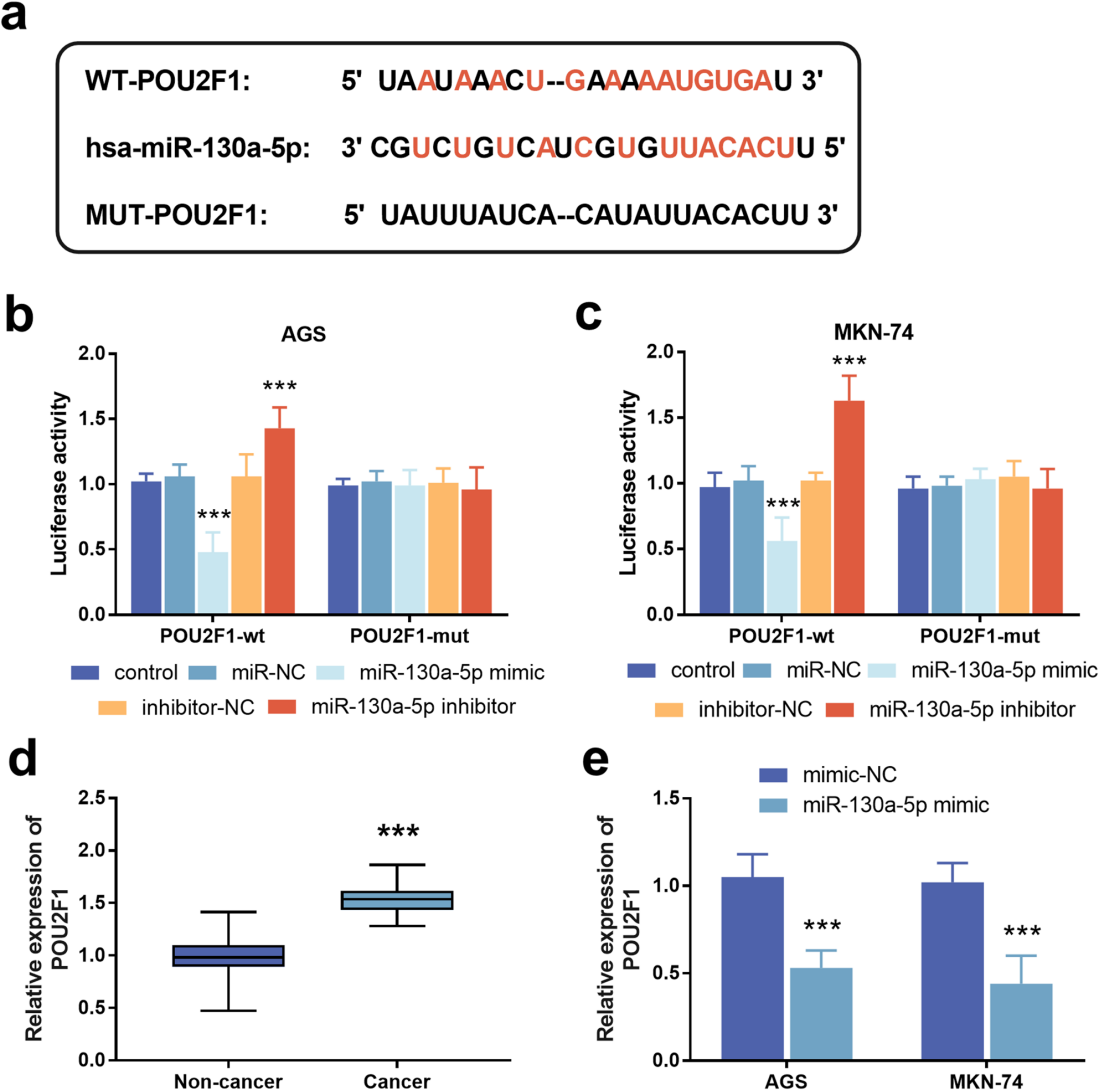
POU2F1 is a downstream target gene of miR-130a-5p. **a**. The complementary site of POU2F1 and miR-130a-5p. **b**-**c**. Dual-luciferase reporter assay. **d**. Expression of POU2F1 in gastric cancer tissues. **e**. Overexpression of miR-130a-5p inhibits the expression of POU2F1

The potential binding sites of POU2F1 and miR-130a-5p are shown in Fig. 7a. The luciferase reporter gene assay clarified the interaction between POU2F1 and miR-130a-5p (Fig. 7b-c, *p* < 0.001). The expression level of POU2F1 in GC tissues is higher than that in non-tumor tissues (Fig. 7d, *p* < 0.001). Overexpression of miR-130a-5p in AGS and MKN-74 cells can significantly down-regulate the expression level of POU2F1 (Fig. 7e, *p* < 0.001).

## Discussion

Among 97 paired GC tissues, RT-PCR verified the elevated expressions of linc01748 in GC tissues, and these findings were in keeping with those in the GEO database and LnCAR database. The overall survival rate of patients with low linc01748 expression was obviously higher than that of patients with high expression. Linc01748, tumor size and infiltration depth were independent factors influencing the survival of GC patients. In cytological studies, linc01748 may affect the function and EMT of GC cells by regulating the miR-130a-5p/POU2F1 axis.

As the high-throughput RNA sequencing technology develops, lncRNA has received unprecedented attention. Researchers are constantly exploring various mechanisms by which it affects gene expression programs and cell functions to reveal its key role in tumorigenesis and development. The expression of lncRNA is tissue-specific, a feature that enables it to play a crucial role in tumorigenesis, diagnosis and treatment [22]. LncRNAs with elevated expression in tumor tissues may play a promoting role in cancer, such as SNHG7 and neuroblastoma, linc00847 and lung cancer [23, 24]. In this paper, the gene expression data of GC tissues and their adjacent non-cancer tissues were analyzed online using the GEO database and the LnCAR database respectively. Both suggested that linc01748 was upregulated in GC tissues. Furthermore, the conclusion that linc01748 has carcinogenic effects is consistent with the research results previously conducted by Tan. This gene is upregulated in non-small cell lung cancer [15]. The antitumor effect of linc01748 potentially determined by the unique repertoire of interacting miRNAs and mRNAs in different cellular environments.

In the GC cell lines AGS and MKN-74, inhibition of linc01748 significantly suppressed cell proliferation, migration, invasion and EMT, indicating that linc01748 played a pro-oncogenic role in GC and promoted the malignant biological behavior of GC cell lines. It has been found that the number of lncRNAs has far exceeded that of protein-coding genes and miRNA genes, and their structures are more diverse [25]. This means that they contain more significant biological implications. Many reports have confirmed that lncRNA is strongly associated with the occurrence and development of tumors [26]. Due to its significant role in carcinogenesis and tumor suppression, it has become a research warm spots in recent years. To date, the ceRNA network mechanism has become one of the most mature mechanisms for studying the interaction between lncRNA and miRNA in the field of cancer research. For example, linc00483 acts as a miR-30a-3p sponge and adsorbs it, thereby releasing the expression of SPAG9 and playing a role in promoting the proliferation and migration of GC cells [27]. TRPM2-AS releases SPP1 through spongy miR-497-5p, thereby promoting the proliferation, migration and invasion of endometrial cancer [28]. In this part of the study, the potential target miRNA of linc01748 in GC was preliminarily predicted through the bioinformatics database. By consulting relevant literature, it was found that miR-130a-5p was lowly expressed in GC. After knocking down linc01748, it was reported that the expression of miR-130a-5p was upregulated in gastric cancer cell lines. This result is consistent with previous bioinformatics predictions and literature review results. Subsequent experimental results also confirmed this regulatory relationship axis.

MiRNA usually inhibits gene expression by binding to the 3’-UTR of mRNA, prevents mRNA translation or accelerates its degradation, thereby functioning as oncogenes or tumor suppressors in cancer [29]. For example, Yang et al. reported that miR-3650, as a carcinogen, drives the proliferation and migration of gastric cancer by directly targeting PTEN to activate the PI3K-AKT-mTOR pathway and inhibit the Hippo pathway [30]. Existing literature has proved that miR-130a-5p is lowly expressed in GC [31]. Xian et al. discovered through in vitro experiments that miR-130a-5p regulates CB1R through the Wnt/β-catenin signaling pathway to participate in the malignant biological behaviors [32]. Similarly, this subject also found that the expression of miR-130a-5p was downregulated in GC and negatively correlated with the expression of linc01748. Subsequently, through the “rescue experiment”, it indicated that simultaneously knocking down miR-130a-5p and linc01748 could partially reverse the “tumor suppressor” effect caused by knocking down linc01748 alone.

POU2F1, also known as octamer-binding transcription factor 1, is encoded by the gene locus on chromosome 1q24.1 [33]. It is a ubiquitous transcription factor that can regulate target gene expression of cell cycle [34]. Previous papers have shown that POU2F1 is highly expressed in liver cancer tissues [35]. There are also studies reporting that POU2F1 is upregulated in GC and is significantly associated with poor prognosis [36]. This study demonstrated an increased expression of POU2F1 mRNA in GC tissues, and the dual-luciferase reporter assay confirmed the interaction between POU2F1 and miR-130a-5p. This study confirmed that the transcription factor POU2F1 is a direct functional target of miR-130a-5p. This discovery positions POU2F1 as a key downstream effector of the Linc01748/miR-130a-5p axis. Even though the current focus of the work is to clarify the upstream regulatory circuit, the combination of existing literature enables us to propose a mechanism framework on how POU2F1 drives the progression of gastric cancer. Previous studies have demonstrated that POU2F1 promotes the malignant behavior of tumor cells by enhancing cell proliferation, aerobic glycolysis and pentose phosphate pathway activity [37]. This provides a reasonable explanation for the proliferation phenotype we observed after overexpression of linc01748. POU2F1 has been confirmed to be associated with the regulation of genes related to epithelial-mesenchymal transition and metastasis [38], which is consistent with our data showing that linc01748 promotes EMT. Altogether, we speculate that the linc01748/miR-130a-5p/POU2F1 axis exerts its carcinogenic effect at least partly by activating a network that can accelerate the cell cycle process. Verifying this complete network from lncRNA to transcription factors and then to global transcriptional changes will be an important direction for our future research.

This study found that linc01748 upregulates the expression of POU2F1 in gastric cancer by adsorbing miR-130a-5p, thereby exerting the function of an oncogene. Our results are consistent with those in the database, jointly supporting the carcinogenic property of linc01748 in gastric cancer. However, our research has expanded functionally, not only verifying its proliferation-promoting effect but also revealing for the first time its strong influence on cell migration and invasion. In terms of the mechanism of action, our discovery is highly novel. Although linc01748 has been reported to regulate other miRNAs through the ceRNA mechanism in non-small cell lung cancer, this study has for the first time established a novel regulatory axis of linc01748/miR-130a-5p/POU2F1 in gastric cancer. This discovery has filled the knowledge gap in this field regarding the unclear specific downstream effector molecules of linc01748 in gastric cancer. The significance of the above findings lies more in their potential clinical translational value. In recent years, individualized treatment based on molecular typing of gastric cancer has become a trend. A highly instructive retrospective clinical study pointed out that, according to the classic Lauren classification, patients with non-intestinal gastric cancer who received adjuvant chemotherapy with the SOX regimen (oxaliplatin + teggio) had significantly better disease-free survival and overall survival than those with the XELOX regimen (oxaliplatin + capecitabine) [39]. This important clinical phenomenon suggests that the intrinsic molecular biological behaviors of different pathological types of gastric cancer may have fundamental differences, thereby leading to different sensitivities to chemotherapy regimens. Therefore, future research efforts will focus on verifying the correlation between the expression of the linc01748/miR-130a-5p/POU2F1 axis and Lauren classification, and further exploring the regulatory role of this axis on the sensitivity of SOX and XELOX protocols in cell and animal models.

This study has limitations. The most important is the relatively small sample size of the clinical cohort. A post-hoc power analysis revealed a statistical power of 66%, which limits the generalizability of our survival findings. Therefore, our clinical results should be interpreted as preliminary and hypothesis-generating. However, the significant *p*-value (0.019) and the strong corroborating evidence from our in vitro functional studies lend credence to the biological plausibility of the linc01748/miR-130a-5p axis in GC. Future studies with larger, prospective cohorts are unequivocally warranted to validate and extend these promising findings.

In conclusion, this study reveals that linc01748 is highly expressed in GC and promotes the malignant biological behavior of GC cells as well as EMT. Subsequent studies further confirmed that linc01748 targets and up-regulates the expression of POU2F1 by binding to miR-130a-5p. This study validated the function and mechanism of linc01748 in GC, providing new targets and basis for the diagnosis and treatment of GC. This study has certain limitations. We have only preliminarily explored the close connection between linc01748 and the biological functions of GC cells, and its association with the prognosis of GC patients. However, the specific mechanism of linc01748 in GC is still unclear and requires further research and exploration.

## Data Availability

The datasets used and/or analysed during the current study are available from the corresponding author, Meini Cen, by < cenmeini96@163.com> from The Affiliated Hospital of Youjiang Medical University on reasonable request.
